# Formulative Study and Characterization of Novel Biomaterials Based on Chitosan/Hydrolyzed Collagen Films

**DOI:** 10.3390/jfb15030069

**Published:** 2024-03-11

**Authors:** Tomás Martínez Rodríguez, Caterina Valentino, Francisco Ramón Rodríguez Pozo, Pablo Hernández Benavides, Francisco Arrebola Vargas, José Manuel Paredes, Claro Ignacio Sainz-Díaz, Guillermo R. Iglesias, Silvia Rossi, Giuseppina Sandri, María del Mar Medina Pérez, Carola Aguzzi

**Affiliations:** 1 Department of Pharmacy and Pharmaceutical Technology, Cartuja Campus, University of Granada, 18071 Granada, Spain; zommart@correo.ugr.es (T.M.R.); pabloj@ugr.es (P.H.B.); mdelmar@ugr.es (M.d.M.M.P.); 2 Department of Drug Sciences, University of Pavia, Viale Taramelli 12, 27100 Pavia, Italy; caterina.valentino01@universitadipavia.it (C.V.); silvia.rossi@unipv.it (S.R.); g.sandri@unipv.it (G.S.); 3 Department of Histology, Institute of Neurosciences, Centre for Biomedical Research (CIBM), University of Granada, 18071 Granada, Spain; fav@ugr.es; 4 Nanoscopy-UGR Laboratory, Department of Physical Chemistry, Unidad de Excelencia en Química Aplicada a Biomedicina y Medioambiente UEQ, Cartuja Campus, University of Granada, 18071 Granada, Spain; jmparedes@ugr.es; 5 Instituto Andaluz de Ciencias de la Tierra, CSIC-University of Granada, 18100 Armilla, Spain; ci.sainz@csic.es; 6 Department of Applied Physics, Faculty of Science, University of Granada, 18071 Granada, Spain; iglesias@ugr.es

**Keywords:** chitosan, hydrolyzed collagen, films, wound healing, mechanical properties, swelling, human keratinocytes

## Abstract

To date, the need for biomaterials capable of improving the treatment of chronic skin wounds remains a clinical challenge. The aim of the present work is to formulate and characterize chitosan (Cs)/hydrolyzed collagen (HC) films as potential biomaterials with improved mechanical and hydration performances compared to single component formulations. Films were made by the solvent casting method, with or without glycerin and/or PEG1500 as plasticizers, resulting in a total of eight formulations. All films were characterized by their physico-chemical characteristics and their mechanical and hydration features. A full factorial design was also used to statistically assess the effect of HC concentration, type and concentration of plasticizers and their possible interactions on mechanical and swelling behaviors. Solid state characterization confirmed the hybrid nature of the films, with suggested electrostatic interactions between Cs and HC. Mechanical and swelling properties, along with the analysis of the experimental design, allowed the identification of formulations containing high HC concentration (2% *w*/*v*) and glycerin or glycerin/PEG1500 as more suitable candidates for skin wound treatment. Finally, viability assay of immortalized human keratinocytes (HaCaT) showed no statistical differences in cell survival compared to the complete culture medium, suggesting their potential as a promising tool for biomedical applications.

## 1. Introduction

The physiological wound healing process consists of four well-coordinated phases [1]. First is the hemostasis phase, in which local vasoconstriction and platelet activation occurs, culminating in a fibrin-rich clot at the site of injury [2]. Subsequently, the inflammatory phase is induced by cytokines released from activated platelets, leading to increased vascular permeability and the arrival of immune system cells at the wound site [1]. Noteworthy is the involvement of M1 macrophages with the ability to secrete pro-inflammatory cytokines (IL-1, IL-8, TNF-α) and oxygen free radicals [3]. The proliferative phase requires the participation of M2 phenotype macrophages to be initiated. These promote the conclusion of the inflammatory phase by releasing anti-inflammatory cytokines (IL-10, IL-13), and secreting both angiogenic and re-epithelialization growth factors to stimulate various proliferative processes [3,4]. Fibroblasts are involved in the extra-cellular matrix regeneration. In response to growth factors, fibroblasts reach the injury site and constitute the granulation tissue by synthesizing and releasing fibronectin, proteoglycans and collagen type III [1]. Once granulation tissue is formed, re-epithelialization begins involving epidermal stem cells and intact keratinocytes from the wound edge moving and settling in the center of the wound. Upon settlement, they regenerate the base membrane and proliferate [5,6]. Angiogenesis involves the formation of new capillaries from vascular endothelial cells of wound blood vessels. This process is triggered by hypoxic conditions that stimulate M2 macrophages and keratinocytes to release pro-angiogenic growth factors [7,8]. Finally, in the remodeling phase, both granulation tissue and excess blood capillaries from angiogenesis are removed. Furthermore, type III collagen is replaced by type I collagen in the granulation tissue, and myofibroblasts achieve complete wound closure [2].

Chronic wounds are mostly defined as those which do not complete the healing process in less than 3 months [5]. This type of wound is responsible for large economic losses, with an estimated healthcare expenditure of 1–3% in developed countries and work-related economic losses associated with reduced productivity and increased absenteeism [9]. It also causes losses in the patient’s quality of life, resulting in a significant decline in personal well-being [10]. Generally, chronic wounds are stalled in the inflammatory phase due to a high neutrophil and M1 macrophage infiltration into the injury. Such behavior could be related to an infection; indeed, chronic wounds often have a microbiome with an excess of pathogenic biofilm-forming bacteria on their surfaces [6]. In addition, the transition to the M2 phenotype macrophage is hindered, blocking the transition to the proliferative phase [4]. Re-epithelialization is impaired, as wound edge keratinocytes are unable to respond to growth factors and suffer from genetic mutations [6,8]. Matrix metalloproteinases excess in the lesion impedes extracellular matrix regeneration by degrading extracellular matrix components and several growth factors [5]. Likewise, fibroblasts are not fully functional and do not respond adequately to growth factors, further hindering the same process [1].

Conventionally, chronic wounds have been treated by monitoring four key aspects: removal of necrotic tissue, infection prevention and treatment, wound moisture management and perilesional skin condition control. Such therapy has several disadvantages. Namely, it is associated with a healing rate of less than 50% [11]. Its application also implies an additional economic burden on the healthcare system due to frequent dressing changes and increased risk of wound infections [10]. In relation to antibiotic use, prolonged administration of antibiotics increases the risk of antimicrobial resistance [12]. It is important to note that conventional dressings serve only as a physical barrier to prevent microbial contamination of the wound. Likewise, some have a limited ability to maintain optimal moisture levels in the lesion [11].

Biopolymeric films have additional properties, in comparison with conventional dressing [13], such as supporting the cells involved in the wound healing process, stimulating the processes of migration, proliferation and differentiation [6], protecting and releasing drugs in a controlled manner, and exhibiting bioactive properties that enhance the healing process [14,15]. They can be prepared by the solvent casting technique, which stands out for its simplicity and inexpensive performance [16]. This technique consists in dissolution of the components in a proper solvent, casting the obtained solution on a flat surface mold and leaving to dry to a solid film [17]. As constituent materials, biopolymers show high biocompatibility, optimal biodegradability and low immunogenic capacity [18]. Moreover, they have recently been used in molecular dynamic simulation studies to design new biomaterials for wound healing applications [19]. Among them, polysaccharides (chitosan, alginates, hyaluronic acid, etc.) and proteins (collagen, fibroin, gelatine, etc.) can be distinguished [15].

Chitosan is a linear polysaccharide composed of D-glucosamine and N-acetyl-D-glucosamine monosaccharides linked by β (1 → 4) bonds [20]. Its biological properties justify its use as a biomaterial in the production of scaffolds for wound healing purposes, as it improves the progression of the different phases of the healing process. In the hemostasis phase, it can promote the formation of a clot at the site of injury [21]. It also has antimicrobial, antioxidant and anti-inflammatory properties, supporting the progression of the inflammatory phase [20,22]. At the proliferative phase, chitosan can stimulate fibroblast activity in the wound area, promoting the deposition of new collagen fibers and hyaluronic acid synthesis [21,23]. In the process of scaffold development, the use of chitosan is advantageous, considering that it can constitute scaffolds in a wide variety of forms [13]. However, chitosan films become too brittle and stiff, needing the addition of plasticizing agents [24].

Hydrolyzed collagen refers to a complex of low molecular weight peptides (3–6 kDa) obtained by native collagen denaturation and hydrolysis [25]. In comparison to native collagen, the use of hydrolyzed collagen has some advantages. It is more soluble in water and does not generate viscous aqueous solutions, allowing the use of larger amounts for therapeutic purposes, is more cost-effective as it has a simpler production process, and has improved organoleptic properties [26]. In vitro, hydrolyzed collagen has proven beneficial properties for wound healing purposes by stimulating chemotactic fibroblast migration, as well as fibroblast and keratinocyte proliferation [25]. However, by itself, it is not able to constitute scaffolds for wound healing purposes, its combination with other polymers that exhibit such capacity being necessary [27]. To date, few supports based on collagen hydrolysates and natural and/or synthetic polymers have demonstrated prominent properties for chronic wound healing [28,29,30].

Given these premises, the aim of this work is the design of chitosan/hydrolyzed collagen film formulations as novel biomaterials to be applied in chronic skin wound healing. Films were prepared by solvent casting technique. Their hybrid (polysaccharide/peptide) nature was expected to enhance the performance of formulations based on a single component. Polyethyleneglycol-1500 and glycerin were also added to reduce film brittleness, and their influence on film behavior (mechanical and hydration properties) was studied in a full factorial experimental design, which also allowed for the identification of the optimal amounts of the components in the formulations. Films were characterized in terms of physico-chemical properties, using FTIR and thermogravimetric techniques, in order to study possible interactions among their components. Preliminary in vitro biocompatibility on human keratinocytes was also assessed as essential requirement for potential biomedical applications of the obtained films.

## 2. Materials and Methods

### 2.1. Materials

Medium molecular weight chitosan (Cs), polyethyleneglycol-1500 (PEG1500), Dulbecco’s Modified Eagle’s Medium–high glucose (4.5 g/mL) (DMEM) and heat-inactivated Fetal Bovine Serum (FBS) were purchased from Merck Life Science S.L.U. (Madrid, Spain). Hydrolyzed collagen (HC) was obtained from Kelisema S.R.L. (Tavernerio, Italy). Glycerin (98.3% purity) (GLY) was acquired from Fagron Ibérica S.A.U. (Barcelona, Spain). Thiazolyl Blue Tetrazolium Bromide (MTT) Biochemica (C_18_H_16_BrN_5_S, M = 414.33 g/mol, 98% purity) was procured from PanReac Applichem ITW Reagents (Barcelona, Spain). All other chemicals and solvents were high-quality analytical grade and used as procured.

### 2.2. Methods

#### 2.2.1. Preparation of Cs/HC Films

For the preparation of Cs and HC based films, Cs solution (2% *w*/*v*) was prepared by dispersing Cs powder in an acetic acid aqueous solution (0.5 M) under continuous magnetic stirring (500 rpm) for 12 h. After this time, Cs solution was centrifuged at 6000 rpm for 30 min to separate possible impurities naturally present in Cs powder (HERMLE Z 322 K Centrifuge, Biogen Cientifica S.L., Madrid, Spain). Aqueous HC solutions (2% *w*/*v* and 4% *w*/*v*) were prepared by dissolving HC powder in distilled water. Then, HC solutions were mixed with Cs solution in a 1:1 weight/weight ratio. Plasticizers (GLY and/or PEG-1500) were slowly added to the resulting Cs/HC blends and kept under magnetic stirring (500 rpm) for 4 h. Finally, 8 mL of the resulting blends were poured into 5 cm diameter silicone molds and were allowed to dry at room temperature under a fume hood (air flow: 0.4 m/s) (film samples F1–F6, Table 1). Formulations without plasticizers were also prepared as references, namely R1 and R2 for low and high HC concentration, respectively. Table 1 reports the final quali–quantitative composition of Cs/HC blends prepared for subsequent film formation after solvent casting.

#### 2.2.2. Characterization of Cs/HC Films

##### Solid-State Characterization

Fourier-transform infrared spectroscopy (FTIR) spectra of the formulated films was obtained using the JASCO 6200 spectrophotometer with SPECTRA MANAGER v2 software and an attenuated total reflectance (ATR) accessory (JASCO Inc., Easton, MD, USA). Measurements were carried out at wave numbers between 400 and 4000 cm^−1^ at a resolution of 0.25 cm^−1^.

Thermogravimetric analysis (TGA) of each film was obtained by means of a METTLER TOLEDO mod. TGA/DSC device with an FRS5 sensor and a precision microbalance (±0.1 μg) (Mettler-Toledo GMBH, Cornellà del Lobregat, Barcelona, Spain). Samples of 10 mg were used for heating in a nitrogen atmosphere at 5 °C/min in the 35–935 °C range.

##### Morphological Characterization

The physical appearance of the films was reported by means of photographs on a uniform black background. The weight of the films was measured after their removal from the silicone mold using an analytical balance with a ±0.1 mg accuracy (Boeco BBI-31 balance, Boeckel and Co. GmbH and Co. KG, Hamburg, Germany). The thickness of the films was assessed using a digital caliper with a measuring range of 0 to 150 mm and a resolution of 0.01 mm (Digital caliper CD-15DAX, Mitutoyo Corporation, Japan). Average thickness was calculated using five different measuring points for each film.

##### Mechanical Properties

The mechanical properties were evaluated by means of a texture analyzer equipped with a 5 kg load cell (TX.AT plus Texture Analyzer, Stable Micro Systems, Catteshall Mill, Catteshall Rd, Godalming GU7 1JW, UK), as described in the literature for thin polymeric film samples [31,32]. Tensile tests were performed on rectangular pieces of each film (3 × 1 cm^2^). Such pieces were placed between two clamp probes (type A/TG) with an initial distance of 1 cm between them. The lifting speed of the upper probe was kept constant at 0.5 mm/s. The final distance between probes was set at 10 cm. Mechanical properties, such as tensile strength (TS) and elongation at break % (EAB %), were determined. TS was calculated considering Equation (1):

(1) $$\mathrm{TS}\;\left(\mathrm{MPa}\right)=\frac{F_{max}}{A}$$

where F_max_ is the maximum force applied (Newtons), and A is the cross-sectional area of the rectangular pieces (m^2^). The cross-sectional area was determined by the thickness and width of the rectangular cut-outs.

EAB % was calculated according to Equation (2):

(2) $$\mathrm{EAB}\;\left(\%\right)=\frac{L-L_{0}}{L_{0}}\;\times \;100\%$$

where L is the distance covered by the probes up to the break of films samples (in mm), and L_0_ is the initial distance between the probes (in mm).

Tensile tests were replicated 6 times for each film formulation.

##### Hydration Properties

Films were immersed in 100 mL of 0.5 M NaOH solution for 30 min to remove any acetic acid residue. Subsequently, films were washed by soaking them in 50 mL of phosphate buffered saline (PBS) solution pH 7.4 for 5 min. The PBS solution was prepared following the European Pharmacopoeia adding a 0.2 M KH_2_PO_4_ solution to a 0.1 M NaOH solution. Finally, films were dried in a desiccator with P_2_O_5_ until a constant weight was achieved. Hydration properties of the films were then determined by evaluating their swelling capacity and wettability.

For the determination of swelling capacity, circular samples of 2.2 cm diameter were prepared from the treated and dried films. These cuttings were weighed to determine their initial weight (w_i_). Then, they were immersed in 8 mL of PBS to keep the pH of the medium constant; at predetermined times, samples were collected, placed on filter paper to remove the excess of water and, finally, weighed (w_t_) with an analytical balance of ±0.1 mg accuracy (Boeco BBI-31 balance, Boeckel and Co. GmbH and Co. KG, Hamburg, Germany). The swelling coefficient (%) of the films was calculated following Equation (3):

(3) $$\mathrm{Swelling}\;\mathrm{capacity}\;\left(\%\right)=\frac{w_{t\;}-w_{i}}{w_{t}}\;\times \;100$$

The determination of the swelling capacity was replicated 3 times for each film.

The wettability of the films, cut in circular samples with a diameter of 1.2 cm, was determined by direct contact angle measurements, following the sessile drop method described in the European Pharmacopeia. For this purpose, an apparatus consisting of a dispensing syringe, a sample plate, a 40× magnification microscope camera (PixeLINK PL-A661, 1.3 Megapixel monochrome FireWire IEEE 1394, Ottawa, ON, Canada) and a white light source was used. The syringe was filled with distilled water, able to pour 5 μL drops onto the surface of the film.

Six replicates were performed for each film.

#### 2.2.3. Full Factorial Experimental Design

To identify, on a statistical basis, the contribution of the formulation components (factors) and their concentrations (levels) on the mechanical (EAB %) and hydration (swelling capacity) properties of the films, a 2^3^ full factorial design was employed. The chosen factors were HC (factor A) and plasticizers (GLY, factor B; PEG1500, factor C), each at two levels (low: −1; high: +1). The resultant experimental design matrix is shown in Table 2. STATGRAPHICS Plus 5.1 software (Statgraphics Technologies Inc., The Plains, VA, USA) was used to carry out the analysis of the experimental design.

#### 2.2.4. Microscopic Analysis

The microstructural images of optimized films were obtained by scanning electron microscopy. The images were taken on a GEMINI (FESEM) CARL ZEISS microscope (Carl Zeiss Meditec Iberia S.A.U.—Medical Solutions, Madrid, Spain) equipped with electron source by Schottky field emission (hot cathode). Samples were coated with gold (NANOTECH SEMPREP2) before they were examined.

#### 2.2.5. In Vitro Biocompatibility Measurements

Cytotoxicity assays of optimized film formulations were carried out on immortalized human epidermal keratinocytes (HaCaT300493, CLS Cell Lines Service GmbH, Eppelheim, Germany), considering their role in wound healing process. HaCaT cells (43rd–45th passages) were cultured in 75 cm^2^ polystyrene flasks with Complete Medium (CM), consisting of Dulbecco’s Modified Eagle’s Medium–high glucose (DMEM) supplemented with heat-inactivated Foetal Bovine Serum at 10% *v*/*v* (FBS). Cells were incubated at 37 °C with 98% relative humidity and 5% CO_2_ atmosphere. After being treated with NaOH 0.5 M and washed with PBS, the films were cut into samples of 5 mm in diameter and subjected to UV-irradiation for 2 cycles of 20 min on each side. Subsequently, 100 µL of cell suspension (at a density of 30,000 cells/cm^2^) were seeded in a 96-well plate together with 100 µL of CM. After 24 h, CM was renewed and film samples were added. HaCaT cells were kept incubated in contact with films for 24 h and 72 h. Nine replicates were performed for each sample; cells in CM were used as positive control. For comparison purposes, assays were also performed on pristine chitosan films at the same concentration as in the optimized formulations (Cs, 1% *w*/*v*). After 24 h and 72 h, films samples were removed using a needle and MTT assay was performed. Briefly, CM was removed, 200 μL of DMEM plus 50 μL of MTT solution (5 mg MTT/mL PBS) were added in each well, and cells were left incubated for 4 h. After 4 h, DMEM with MTT was removed and 200 μL of DMSO together with 25 μL of Sörensen’s glycine buffer were added. After 15 min shaking at 100 rpm (Rotamax 120, Heidolph, Germany), the optical density was read at a wavelength of 562 nm (Universal Microplate Reader ELx800, Bio-Tek Instruments Inc., Santa Clara, CA 95051, USA).

#### 2.2.6. Statistical Analysis

One-way ANOVA (analysis of variance) together with Scheffé’s *post hoc* test for statistical analysis of the experimental groups were performed on the obtained data, using SPSS 28 software (IBM^®^, New York, NY, USA). Differences were significant at *p* values less than 0.05.

## 3. Results and Discussion

### 3.1. Characterization of Cs/HC Films

#### 3.1.1. Solid-State Characterizations

**Figure 1:**
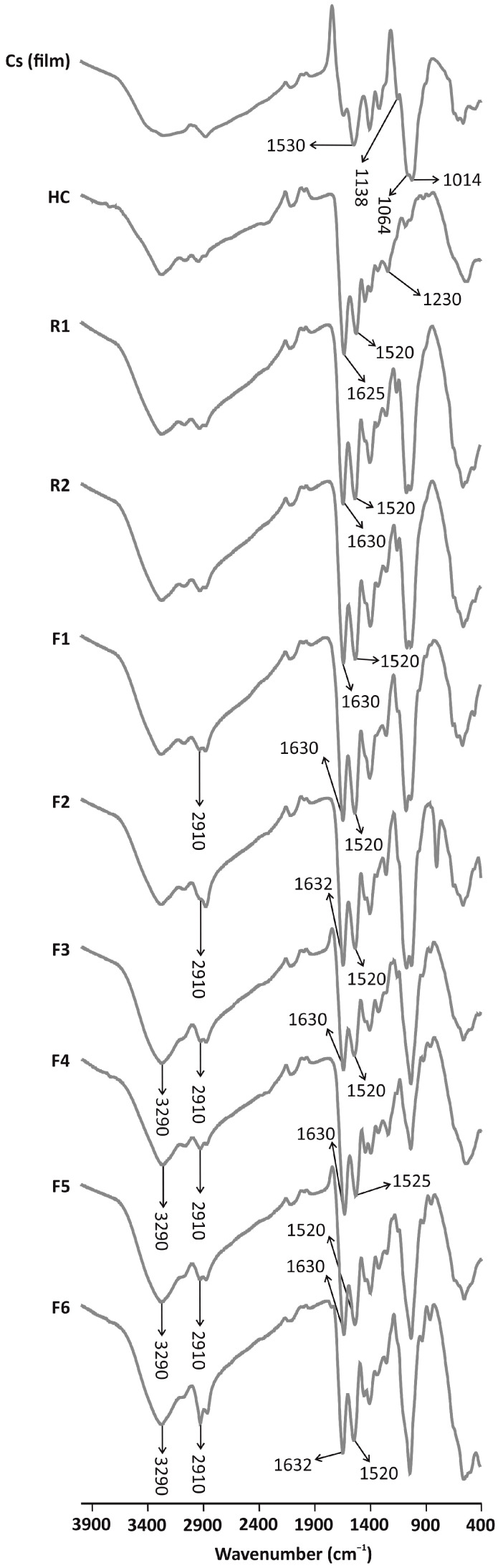
FTIR spectra of the film formulations and their pristine components.

Figure 1 shows the FTIR spectra of the films and their pristine components. Concerning Cs, bands at 1625 cm^−1^, associated with C=O stretching vibrations of amide II group, and at 1530 cm^−1^, related to N-H bending vibrations of amide II group, are distinguished. These values are consistent with those found in the literature for Cs acetate and are due to the interaction between amide II and acetic acid in solution [33,34]. Bands at 1138, 1064 and 1014 cm^−1^ appear due to the saccharide structure of chitosan (e.g., the band at 1064 cm^−1^ involves the C-O bridge of the glucosamine structure) [35,36]. HC shows characteristic bands at 1625 cm^−1^, corresponding to stretching C=O vibrations of the amide I group [37], and at 1520 cm^−1^ and 1230 cm^−1^, corresponding to amide II and III groups, respectively, which are the result of bending N-H and stretching C-N vibrations [38,39].

The hybrid nature of the films is proved by the presence of distinctive bands of both Cs and HC in spectra. Characteristic bands of Cs are shown at around 1520 cm^−1^ and between 1100–1000 cm^−1^. The presence of HC is verified by the bands at 1630 cm^−1^. The incorporation of plasticizers (Films 1–6) leads to an increase in the intensity of bands related to stretching O-H and C-H vibrations (3290 cm^−1^ and 2910 cm^−1^). The interaction between Cs and HC is confirmed by a slight shift of the amide II band at 1530 cm^−1^ in the Cs film to 1520 cm^−1^ in the Cs/HC films. Additionally, its intensity increases when compared to the pristine components, indicating an interaction between the ammonium groups of Cs and the carboxylates of HC.

**Figure 2:**
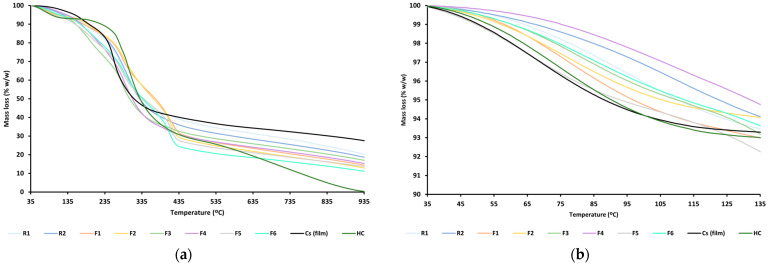
TGA curves of the film formulations and their pristine components: complete curves (**a**) and detail from 35 °C to 135 °C (**b**).

Thermogravimetric analyses performed on the films (TGA curves) are shown in Figure 2. For comparison purposes, the curves of the pristine components (Cs film and HC) are also plotted. The TGA curve of Cs film displays a 3-step process with a total mass loss of approximately 73% (*w*/*w*) (Figure 2). First step (35 °C–135 °C) results in approximately 4% (*w*/*w*) mass loss ascribed to the loss of weakly adsorbed water [40]. Second step (150 °C–200 °C) can be associated with the degradation of acetic acid residues, causing a mass loss of 8.7% (*w*/*w*). Third step (200 °C–930 °C) involves chitosan degradation, which results in a mass loss of 60.2% (*w*/*w*). Such degradation is mainly due to Cs deacetylation and cleavage of glycosidic bonds [41]. A slower degradation process is observed in the range from 400 °C onwards, matching with the pyrolysis of chitosan’s pyranose rings [42]. In the case of HC, the TGA curve reveals a total mass loss of 99.8% (*w*/*w*) with 3 steps in the following ranges: 35 °C–135 °C, 172 °C–490 °C, and 490 °C onwards. First step corresponds to the loss of adsorbed water in the analyzed sample, with a mass loss of 7.3% (*w*/*w*). The next steps may correspond to the loss of hydroxyl groups and water molecules absorbed in HC [43], as well as to the HC combustion process. Both processes involve a mass loss of 92.5% (*w*/*w*).

For all films, the percentage of total degradation was calculated following the rule of mixtures [44], considering the residual masses at 935 °C and water content in % *w*/*w* (Table 3). The results show that the films have a total mass loss between the two main components (69.21% *w*/*w* for Cs film and 92.77% *w*/*w* for HC), corroborating the hybrid Cs/HC character of the films.

#### 3.1.2. Morphological Characterization

**Figure 3:**
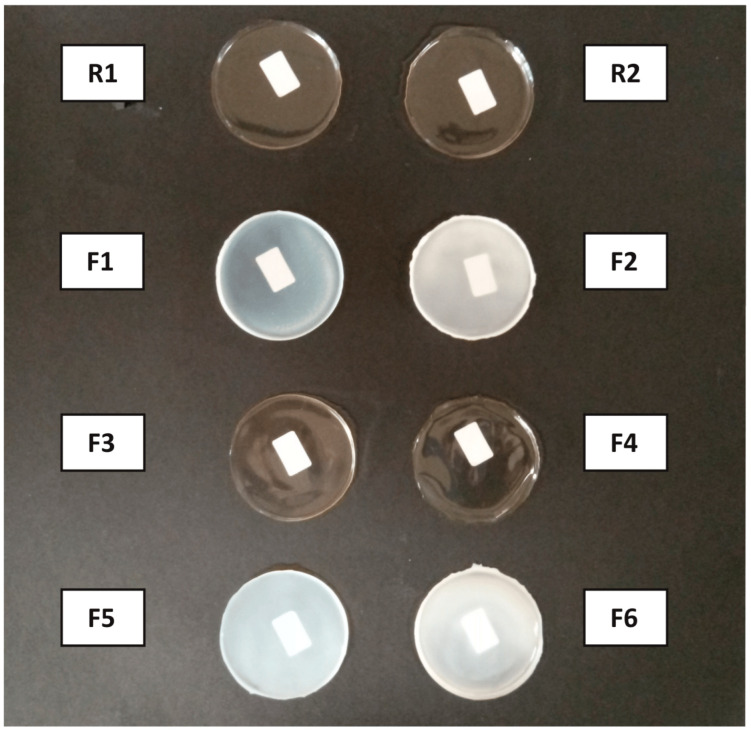
Physical appearance of the films.

Figure 3 shows the films photographed on a black background. A white label was placed behind the films to appreciate the differences in terms of transparency. Reference formulations (R1 and R2) and films containing exclusively GLY as plasticizer (F3 and F4) are more transparent than films incorporating PEG1500 in their formulation (F1, F2, F5 and F6), which show as opaquer and with a more whitish color.

The results of the morphological characterization in terms of thickness and weight are summarized in Table 4.

Plasticizer incorporation leads to an increase in film thickness in comparison with the reference formulations (R1 and R2). These outcomes are consistent with the findings of other studies on the effect of the addition of various plasticizers in chitosan-based film formulations upon thickness [45,46]. In addition, PEG1500 notably increases film thickness compared to GLY. According to Ibrahim et al. [45], this may be due to the plasticizer’s higher molecular weight and a longer chain length. Such features would increase the distance between the biomolecule chains in the film structure. As for film weight, it increases along with the number of components and their relative concentrations. In particular, higher weight values are observed in films containing the highest (2% *w*/*v*) HC concentration. The addition of a single plasticizer (GLY or PEG1500) also leads to increases in film weight, compared to R1 and R2 formulations (F1 and F3 vs. R1; F2 and F4 vs. R2). Further increases can be observed when both GLY and PEG1500 are present, resulting in weight increments of 38% (R1 vs. F5) and 43% (R2 vs. F6) for low and high HC concentrations, respectively.

#### 3.1.3. Mechanical Properties

Films were characterized in terms of TS and EAB % and the results are shown in Table 5. It is known that films must show adequate mechanical strength to maintain their physical integrity and withstand the application of external forces during their placement on wounds. Considering the obtained results, F4 and F6 formulations present adequate TS and EAB % values for skin application [18,47]. Film formulations should also be flexible enough to adapt to the wound surface and resist bending movements which entail the torsion of their structure [18]. Table 4 shows that films with lower HC concentration and a single plasticizer (F1 and F3) show higher TS values, with significant differences compared to their corresponding formulations with 2% (*w*/*v*) HC concentration (F2 and F4). This could be due to the performance of plasticizers and the increase in their relative amounts in F2 and F4. As it has been described, plasticizers decrease film fragility and increase molecular chain movement, resulting in a TS decrease and an EAB % increase [23]. As for EAB %, only significant increases (*p* < 0.05) are observed due to GLY, showing increments of 62.87% (F3) and 67.08% (F4) compared to their respective reference films (F3 vs. R1; F4 vs. R2). Such increments are less prominent for F5 and F6 (47.89% and 36.79%, respectively), being attributable to PEG1500 addition, as this barely shows any improvement in EAB % when used as a plasticizer alone (F1 and F2). Indeed, EAB % deterioration has been described in chitosan-based films with the presence and increase in relative amounts of PEG [48,49].

#### 3.1.4. Hydration Properties

**Figure 4:**
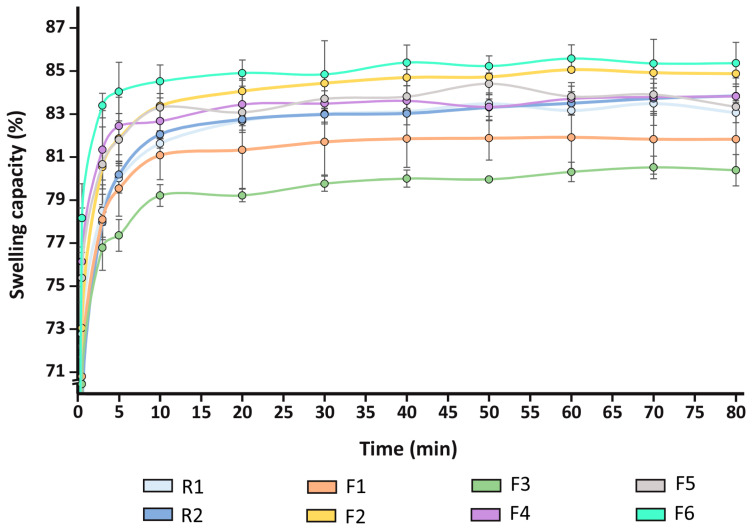
Film swelling capacity profiles (mean values ± s.d.; n = 3).

Chronic wounds may suffer a profuse exudate, slowing healing by damaging perilesional skin [22]. Therefore, film dressings should exhibit adequate water absorption (swelling) capacity to remove excessive moisture and maintain optimal hydration conditions at the wound site [50]. Film swelling capacities over time are shown in Figure 4, where the majority of the formulations reach maximum values of 80–85% after approximately 10 min in PBS. Such values are higher compared to other formulations made from chitosan and native collagen, either in the form of films [51,52] or sponges [53].

Film swelling capacities when equilibrium is achieved are plotted in Table 6. An increase of the swelling capacity with the incorporation of the plasticizers and the augmentation of HC concentration can be distinguished (F2, F4 and F6 vs. F1, F3 and F5, respectively). The presence of both plasticizers (F5 and F6) also leads to a net improvement of the swelling capacity, compared to corresponding formulations that contain only GLY or PEG. In particular, F5 attains increments of 2.31% and 3.79%, compared to F1 and F3, respectively, and F6 shows increases of 0.86% vs. F2 and 1.91% vs. F4, respectively. These results reinforce the idea that the film swelling capacity improves after the incorporation of the plasticizers and increasing HC concentration. Table 6 also shows the contact angles obtained from the studied formulations. For all cases, values lower than 90° can be observed, suggesting that all the films exhibit a hydrophilic surface. Furthermore, in accordance with other studies, a decrease in the contact angle values with the incorporation of both plasticizers is observed [23]. Specifically, there is a reduction from about 80° (R1 and R2) to values ranging from 74° (F1) to 65° (F5). This fact may be due to the hydrophilic and hygroscopic nature of the plasticizers integrated in the film structure [54].

### 3.2. Full Factorial Experimental Design

The estimated effects of the studied factors and their interactions on the response variables (EAB % and swelling capacity), as well as *p*-values resulting from the analysis of the experimental design, are reported in Table 7. Effects with a minus sign indicate that the response decreases as the factor level increases, while plus signs indicate that the response increases as the factor level increases.

For EAB %, both plasticizers have significant, but opposite effects. Specifically, GLY exhibits a high significant (*p* = 0.000) positive effect, which indicates an increase of EAB % with increasing GLY concentration. In the case of PEG1500, an apparent negative effect is shown. However, the analysis indicates a *p*-value at the limit of significance, and therefore its effect can be considered negligible. Concerning swelling capacity, significant positive effects are observed for HC and PEG1500.

**Figure 5:**
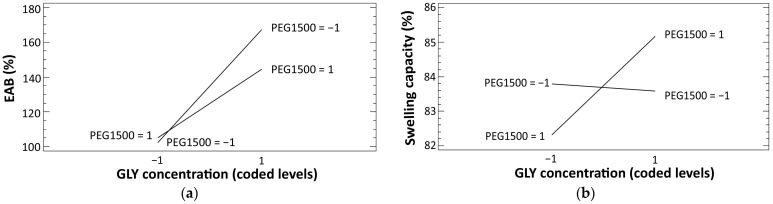
Interaction plots between GLY and PEG1500 for EAB % (**a**) and swelling capacity (**b**).

Moreover, for both responses, the analysis of the experimental design shows that a significant interaction has occurred between the two plasticizers (BC). To better understand their effect, such interactions are shown in detail in Figure 5a,b, for EAB % and swelling capacity, respectively.

Concerning EAB % (Figure 5a), the interaction plot shows that the effect of GLY on the response is higher (steeper line) when the concentration of PEG1500 is lower (coded level: −1). In contrast, regarding the swelling capacity (Figure 5b), the effect of GLY is higher when PEG1500 concentration is higher (coded level: 1).

**Figure 6:**
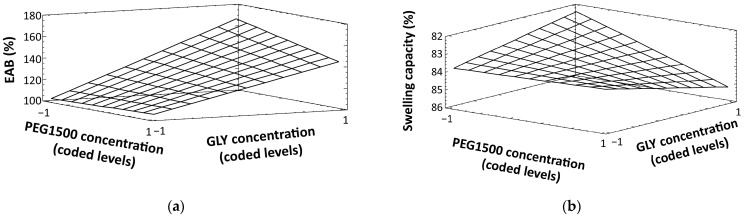
Response surface plots for EAB % (**a**) and swelling capacity (**b**), obtained from the analysis of the full factorial design (response values from all the studied film formulations are given in the plots).

This analysis corroborates the findings described in the characterization of mechanical properties and can be confirmed by the surface response plots (Figure 6). For EAB % (Figure 6a), the response plot shows that higher values (around 170%) are achieved with increasing GLY and decreasing PEG1500 concentrations. This result corresponds to formulations F3 (EAB ≅ 165%) and F4 (EAB ≅ 170%), which contain only glycerin as the plasticizer. In the case of swelling capacities (Figure 6b), higher responses are observed when both plasticizers are present at their high levels (F5 and F6). This is especially evident in F6, which achieves the highest swelling capacity values (≅86%) and contains the highest concentration of HC, as observed in the characterization of hydration properties.

### 3.3. Microscopic Analysis

**Figure 7:**
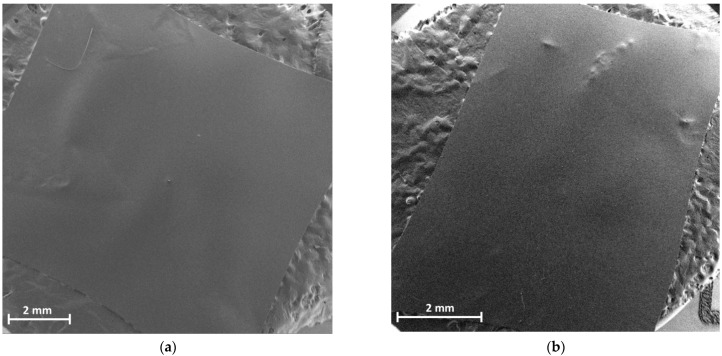
SEM microphotographs of F4 (**a**) and F6 (**b**) film samples.

The microstrucural images of F4 and F6 film formulations are shown in Figure 7. Both samples exhibit smooth and extended surfaces with no sign of pores or roughness patterns, confirming their homogeneous and transparent features.

### 3.4. In Vitro Biocompatibility Measurements

**Figure 8:**
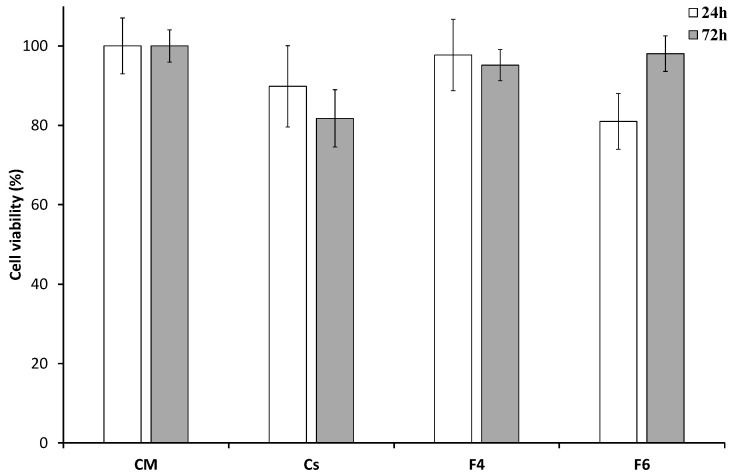
Viability of HaCaT cells after 24 h and 72 h of incubation with F4, F6 and pristine Cs films; CM was considered as control (mean values ± e.s.; n = 9).

Based on results of the full factorial design, F4 and F6 formulations were selected for cell biocompatibility assays. Figure 8 shows the HaCaT cell viability obtained by MTT analysis after up to 72 h of incubation with the studied samples. After 24 h, F4 exhibits significantly higher biocompatibility compared to F6 and pristine Cs. This value is maintained by the F4 film after 72 h and has proven not to be significantly different from cell viability in complete medium (CM). F6 also shows good compatibility with HaCaT cells, increasing their viability from 24 h to 72 h (*p* < 0.001), without statistical difference compared to cells in CM. In contrast, Cs tends to decrease cell viability compared with control. Moreover, after 72 h no significant differences in cell viability were observed after contact with F4 and F6, indicating that both plasticizers do not affect the biocompatibility of the formulations.

## 4. Conclusions

The purpose of this study was to design Cs/HC films with/without plasticizers as novel biomaterials with improved properties in the management of chronic skin wounds, compared to single Cs or HC formulations. Morphological characterization shows that transparent, thin and homogeneous Cs/HC films have been successfully prepared by solvent casting technique. Their preparation is easy and straightforward and does not require additional toxic chemical agents. Solid state characterization carried out by TGA has confirmed the Cs/HC hybrid nature of the films. Van der Waals interactions between Cs (NH_3_^+^ groups) and HC (COO^−^ groups) have been suggested by FTIR spectroscopy. Mechanical and swelling properties, along with the analysis of the experimental design, have allowed the identification of formulations 4 and 6, both containing high HC concentration (2% *w*/*v*), as more suitable for potential application in chronic skin wounds. F4, with glycerin as single plasticizer, is noteworthy given the coincidence of its mechanical properties with reference values reported in the literature for films to be applied on the skin. F6, containing both glycerin and PEG1500, in addition to having acceptable mechanical properties, stands out for its higher swelling capacity (85%) compared to other formulations made from chitosan and native collagen. This also reinforces the finding that film swelling capacity improves by incorporation of the plasticizers and increasing HC concentration. SEM images show smooth and extended film surfaces, indicating successful uniform and transparent features. In addition to having suitable morphological, mechanical and hydration properties, both F4 and F6 films show high biocompatibility with HaCaT cells, which are considered as a reliable in vitro model to evaluate the inflammatory and repair response of human keratinocytes. In future stages, these formulations will be used as supports to incorporate nanohybrids containing antimicrobial agents and growth factors, aiming at the design of advanced and synergistic therapeutic systems for the improved treatment of chronic skin lesions.

## Figures and Tables

**Table 1:** Quali–quantitative composition of Cs/HC blends.

Film	Cs (% *w*/*v*)	HC (% *w*/*v*)	PEG-1500 (g)	GLY (g)
R1	1	1	-	-
R2	1	2	-	-
F1	1	1	0.4	-
F2	1	2	0.6	-
F3	1	1	-	0.4
F4	1	2	-	0.6
F5	1	1	0.4	0.4
F6	1	2	0.6	0.6

**Table 2:** Experimental design matrix.

Films	Factors (Coded Levels)					
	A: HC (% *w*/*v*)		B: GLY (g)		C: PEG1500 (g)	
R1	1	(−1)	-	(−1)	-	(−1)
R2	2	(1)	-	*(−1)*	-	(−1)
F1	1	(−1)	-	(−1)	0.4	(1)
F2	2	(1)	-	(−1)	0.6	(1)
F3	1	(−1)	0.4	(1)	-	(−1)
F4	2	(1)	0.6	(1)	-	(−1)
F5	1	(−1)	0.4	(1)	0.4	(1)
F6	2	(1)	0.6	(1)	0.6	(1)

**Table 3:** Water content and total degradation values of films and pure components.

Film	Residual Masses (% *w*/*w*)		Water Content (% *w*/*w*)	Total Degradation (% *w*/*w*)
	135 °C	935 °C		
R1	93.47	19.77	6.53	73.68
R2	94.11	18.32	5.89	75.79
F1	92.98	14.09	7.02	78.89
F2	94.06	12.63	5.94	81.43
F3	93.16	16.76	6.84	76.4
F4	94.74	15.06	5.26	76.68
F5	92.23	13.37	7.77	78.86
F6	93.61	10.87	6.39	82.74
Cs	96.52	27.31	3.48	69.21
HC	93	0.23	7	92.77

**Table 4:** Thickness and weight of the prepared film (mean values ± s.d.; n = 6).

Films	Thickness (μm)	Weight (mg)
R1	81.17 ± 9.85	197.83 ± 3.51
R2	126.83 ± 16.96	278.16 ± 2.12
F1	98.17 ± 11.49	241.32 ± 5.41
F2	152 ± 13.05	351.48 ± 5.63
F3	86.67 ± 12.34	217.08 ± 2.14
F4	123 ± 10.53	333.6 ± 4.84
F5	99.17 ± 8.20	274.13 ± 7.64
F6	146 ± 11.41	396.4 ± 6.31

**Table 5:** Mechanical properties of the prepared films (mean values ± s.d.; n = 6).

Films	TS (MPa)	EAB (%)
R1	26.86 ± 2.83	102.04 ± 0.17
R2	26.42 ± 5.01	102.45 ± 1.27
F1	31.65 ± 1.45	107.27 ± 2.89
F2	16.30 ± 2.19	103.04 ± 0.96
F3	28.45 ± 3.95	164.91 ± 6.41
F4	16.82 ± 0.86	169.53 ± 23.02
F5	14.61 ± 3.75	149.93 ± 17.62
F6	19.91 ± 3.79	139.24 ± 6.41

**Table 6:** Swelling capacity values once equilibrium is achieved and contact angle values.

Films	Swelling Capacity (%)	Contact Angle (°)
	(Mean Values ± s.d.; n = 3)	(Mean Values ± s.d.; n = 6)
R1	83.67 ± 0.26	80.4 ± 0.9
R2	83.9 ± 0.56	80.5 ± 2.2
F1	82.09 ± 1.35	74.4 ± 2.1
F2	85.06 ± 1.35	66.4 ± 5.1
F3	80.62 ± 0.53	66.2 ± 8.1
F4	84.01 ± 0.71	73.4 ± 4.6
F5	84.41 ± 0.51	65.2 ± 2.5
F6	85.92 ± 0.62	70.8 ± 2.2

**Table 7:** Estimated effects of each factor and their interactions on each response variable.

	EAB (%)		Swelling Capacity (%)	
	Estimated Effect	*p*-Value	Estimated Effect	*p*-Value
A: HC	−2.47	0.5925	2.02	0.0002
B: GLY	52.2	0.0000	0.06	0.8892
C: PEG1500	−9.87	0.0454	1.32	0.0059
AB	−0.57	0.9021	0.43	0.3129
AC	−4.98	0.2872	0.21	0.6147
BC	−12.77	0.0128	1.53	0.0021

## Data Availability

The original contributions presented in the study are included in the article, further inquiries can be directed to the corresponding authors.
